# The Influence of Cultural And Gender Roles On Child Abuse Among Immigrant Communities: A Scoping Review

**DOI:** 10.1007/s40653-026-00936-w

**Published:** 2026-07-01

**Authors:** Abdulbasit Seid, Hang Minh Thai, Titus S. Olorunnisola

**Affiliations:** 1 Action Research Centre, Craigieburn, VIC 3064 Australia; 2 Murdoch Children’s Research Institute, Melbourne, Australia; 3 School of Public Health and Preventive Medicine, Monash University, Melbourne, Australia; 4 Whitley College, University of Divinity, Melbourne, Australia

**Keywords:** Child abuse, Immigrants, Culture, Gender, Impacts

## Abstract

Child abuse has increasingly received attention as a significant global public health and human rights concern. Although culture plays a significant role in child abuse, there are limited studies investigating the role of culture in childhood abuse among immigrant communities. Therefore, the purpose of this scoping review was to synthesize the evidence on cultural issues, including gender-related issues and child abuse, and explore how these cultural factors impact the outcomes of abuse. OVID Medline, OVID Embase, and OVID PsycINFO were searched comprehensively to retrieve eligible articles using predefined search terms that included gender or gender role and culture or cultural role and child abuse and witness or victimisation or perpetration and immigrant or culturally and linguistically diverse and related terms to locate articles. Additional articles were identified through manual searches. Of the 1366 records screened by title and abstract, 18 articles were included in the review. Among included studies reporting on different aspects of child abuse, the prevalence of physical violence ranged from 23 to 78%, while emotional abuse, sexual abuse and neglect ranged 5.2–19.1%, 3.9–13.7%, and 4.7–29.6% respectively. The findings indicated the existence of diverse practices of child physical abuse within the community. Furthermore, patriarchal social norms and cultural acceptance of child abuse were factors that are reported to increase the risk of child abuse. However, the relationship between acculturation and child abuse was inconsistent. Likewise, experiencing child abuse was related to negative mental wellbeing, perpetration of violence and failure to disclose abuse and seek support. Overall, the review identified substantial levels of child abuse among immigrant communities. Different cultural beliefs and gender-related norms were reported to influence the acceptability of child abuse and help-seeking behaviours among victims. To combat this issue, it is crucial to address cultural beliefs that condone child abuse through education and training. Additionally, the provision and enhancement of culturally sensitive services are vital to promote disclosure, prevent negative mental well-being, and break the cycle of violence within the communities.

**Supplementary Information:**

The online version contains supplementary material available at https://doi.org/10.1007/s40653-026-00936-w.

## Introduction

Violence against children is a significant global public health and human rights concern. Globally, about three in four children between 2 and 4 years old experience physical punishment and emotional violence at the hands of their parents/caregivers, while one in five women and one in thirteen men experience sexual abuse as a child (WHO, 2020). Violence during childhood is considered one of the biggest hurdles in achieving sustainable development goals (United Nations, 2015). It has several lifelong deleterious effects on the mental and physical health of the victims, school performance, and social functioning and further reduces productivity at individual and national levels (Currie & Widom, 2010; Elizabeth & Charles, 2020).

The United Nations member states made their commitment to tackling violence against children by upholding children’s human rights as stipulated in the United Nations Convention on the Rights of the Child (UNCRC)(UN General Assembly, 1989). However, due to cultural differences in what makes violence, political commitment, and lack of resources, the implementation of violence prevention policies varies across countries (Daro, 2006; Tobin & Cashmore, 2020). In the contemporary world, culture is no longer limited to certain geographical boundaries as rapidly changing demographic trends and relationships among people across the globe bring differing cultural practices and traditions into contact (Norris & Inglehart, 2009).

Migration has a considerable share in bringing diverse people together and contributing to population dynamics by forming cultural plurality (Esses, 2018). Immigrants, regardless of the reason for immigration, are expected to learn and then adapt to the social and cultural framework of the host societies (Bhugra & Cochrane, 2001). In contrast, they also have distinct and culturally embedded beliefs that shape their practices in various issues (Bhugra & Becker, 2005), including parenting. Parents from immigrant communities often hold parenting goals and expectations derived from their own culture, which might sometimes be inconsistent and considered abusive in the host country (Bornstein, 2012). Parenting difficulties, acculturation problems, and related mental health issues are common challenges immigrants face, which can increase the likelihood of child abuse (Bhugra, 2004; Bhugra & Ayonrinde, 2004; Wu & Chao, 2011). Moreover, people from immigrant communities may hold different norms regarding the family structure that contribute to child abuse. For example, in patriarchal societies where women and girls are devalued, their rights are disregarded, and the well-being of victims is often secondary to the perpetrator’s reputation (UNICEF, 2020), leaving girls vulnerable to abuse and discouraging them from seeking help.

While previous studies have explored the possible role of culture (Stoltenborgh et al., 2011), including gender-related norms on child abuse, these studies were largely based on the general population living in different countries and/or cultural contexts. Therefore, there is a need for further studies to understand the specific cultural issues contributing to child abuse among immigrant communities and how these cultural issues impact the outcomes of abuse. Hence, this scoping review aimed to synthesize the evidence on cultural issues, including gender-related issues and child abuse, and explore how these factors impact abuse outcomes.

## Review Questions

1. What cultural issues, including gender-related contexts, were reported in relation to the childhood experience of domestic violence among immigrant communities?
2. How do cultural issues, including gender-related issues, impact the outcomes of abuse during childhood among people from immigrant communities?

## Inclusion Criteria

### Population

The review included research reported on child abuse within immigrant communities. This included studies that reported on personal accounts of childhood abuse from individuals who were victims, perpetrators, or caregivers of children who experienced abuse across various cultural contexts. Additionally, studies that focused on the influence of gender and cultural roles on childhood abuse within immigrant communities were also considered for inclusion in the review.

### Concept

In this review, child abuse is defined as experiencing one or more of the following before the victim turned 18 years old: physical violence (including corporal punishment), sexual and psychological/emotional violence, and neglect, consistent with previous definitions (UNICEF, 2020).

### Context

All people from immigrant communities, regardless of their geographical area, were included in the review.

### Types of Studies

The review considered all studies reported on culture (including acculturation) and gender (including gender-related norms, perpetrators, and victims’ gender) in relation to child abuse among people from immigrant communities. Studies employing quantitative, qualitative, and mixed-methods designs were included. However, studies that did not clearly indicate the immigration background of the population, those that reported on domestic violence in general without emphasizing the cultural element of child abuse or gender role, those that reported on internally displaced people, and studies published in a language other than English were not included.

## Methods

The review adhered to JBI (the Joanna Briggs Institute) guidelines for the conduct of the study (Peters et al., 2020). To ensure comprehensive reporting, the review was guided by the Preferred Reporting Items for Systematic Reviews and Meta-Analyses extension for Scoping Reviews (PRISMA-ScR) (Tricco et al., 2018). OVID Medline, OVID Embase, and OVID PsycINFO were searched comprehensively to retrieve eligible articles using predefined search terms. The following key search terms with their related terms were used to identify the articles: (gender or gender role or female or male and (culture or cultural role) and (child abuse or domestic violence or child physical abuse, child sexual abuse or child emotional abuse or child psychological abuse, child neglect or family violence) and (witness or exposure or victimization or perpetration or experience) and (immigrant or culturally and linguistically diverse or cultural diversity or born overseas or refugee or foreign-born or non-English speaking backgrounds or ethnic minorities). All three databases were searched from their respective inception dates to 20 August 2022, with the searches updated on 5 June 2026. In addition, reference list searching of included studies was conducted to identify further relevant articles.

All articles identified through the search were exported to endnote V20, and then the duplicates were removed. Two independent reviewers screened articles by title and abstract, followed by full text screening against the inclusion and exclusion criteria. Any disagreements were resolved through discussion or consultation with a third reviewer. We did not conduct a quality assessment for the included studies.

Data extraction was conducted by using a standardized pre-piloted data extraction tool by two independent researchers. Then, the two independent researchers came together and checked all extracted data to make sure that all necessary information was extracted correctly. The following information was extracted from the eligible studies: author(s), year of publication, country of origin, types of abuse studied, target population, study type, aim/purpose, population/sample size, study tool used, and key findings.

The included studies were summarized using tables, and a narrative summary was provided as per the objectives of the review. First, characteristics of the studies were reported by country of origin, study design, year of publication, and types of abuse studied. The relevant statistical findings from the quantitative studies were reported to provide general insight into the magnitudes of different types of abuse. Furthermore, narrative reviews were conducted to synthesize the qualitative studies by identifying themes that coincide with quantitative findings. Three themes were identified and described in detail: (1) cultural beliefs, disciplinary practices, and acculturation as contexts for child abuse; (2) gender roles, family power dynamics, and disclosure of abuse; and (3) the impacts of child abuse.

## Results

**Fig. 1:**
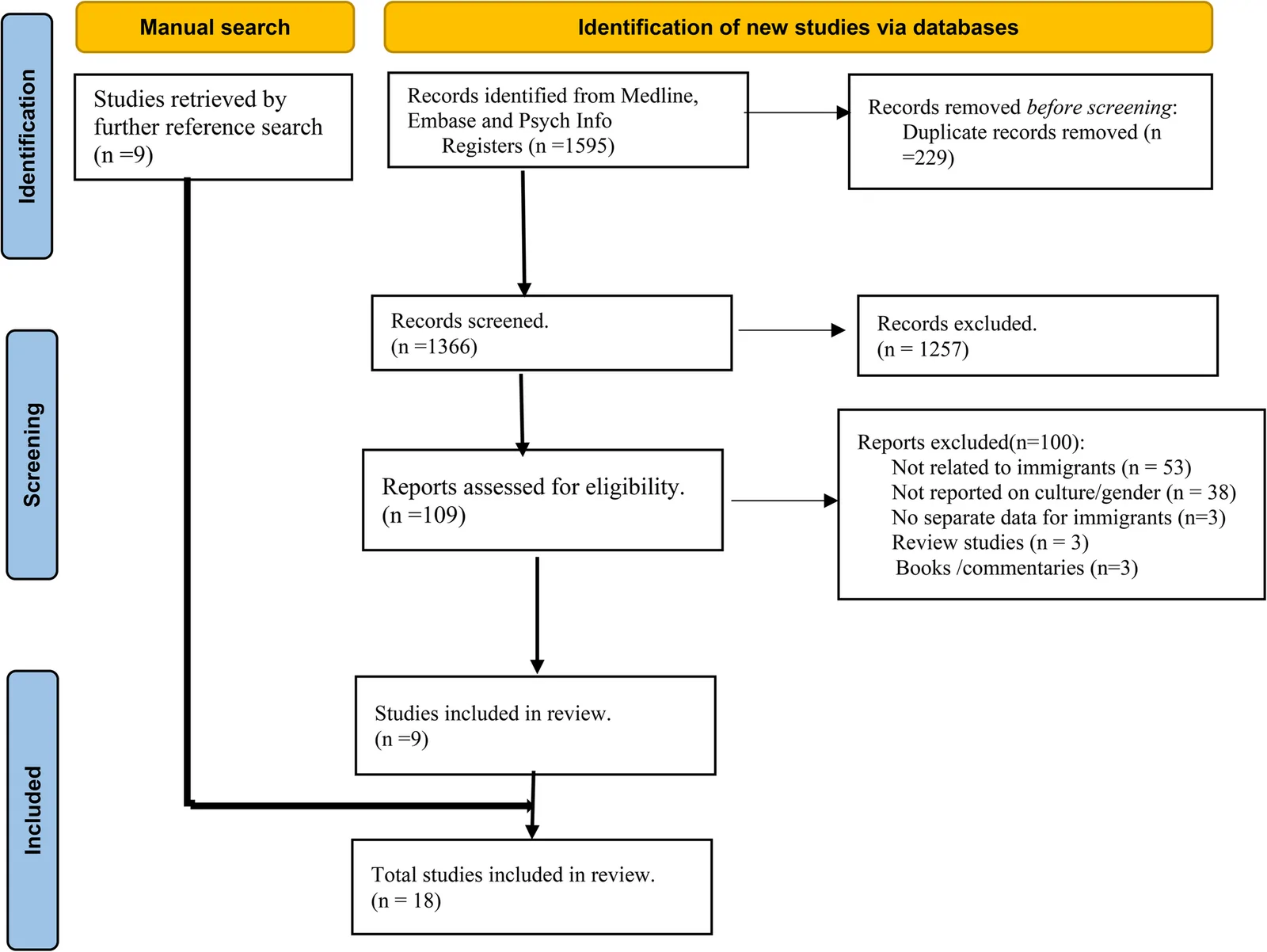
PRISMA flow diagram for scoping review which included database and manual searches

A total of 1,595 articles were retrieved from the three databases. Of those retrieved articles, 229 duplicates were removed. The remaining 1366 articles were screened using titles and/or abstracts. One hundred and nine articles were considered for further full-text review, while the remaining 1257 were excluded. Of 109 publications considered for the full review, nine were eligible to be included in the study. As the database searches identified only a limited number of eligible studies, reference list searching was undertaken, yielding an additional nine articles. In total, 18 articles were included in the review (Fig. 1).

### Characteristics of the Included Studies

A total of 65,377 respondents in 18 studies were included in the review. Of all included studies, nine were from the USA (Black et al., 2013; Dettlaff & Johnson, 2011; Kimber et al., 2015; Kohli & Feneran, 2020; Maker et al., 2005; Park, 2001; Rai et al., 2022; Rhee et al., 2012; Tajima & Harachi, 2010), and two each were from Australia (Fisher, 2013; Renzaho et al., 2011) and the UK (Gilligan & Akhtar, 2005; Widanaralalage et al., 2025). Of the remaining five studies, one included participants from both the USA and South Korea, and one study each was conducted in Scotland, the Netherlands, Norway, and Finland. All included studies were conducted from 2001 to 2025. Eleven studies used quantitative methods (Black et al., 2013; Dettlaff & Johnson, 2011; Kimber et al., 2015; Lee et al., 2014; Maker et al., 2005; Park, 2001; Rai et al., 2022; Rhee et al., 2012; Skafida et al., 2022; Sunley et al., 2020; Tajima & Harachi, 2010) to answer research questions while the remaining seven studies were qualitative.

Regarding target population, eight studies reported on parents and/children (Dettlaff & Johnson, 2011; Filio Degni et al., 2006; Kohli & Feneran, 2020; Park, 2001; Renzaho et al., 2011; Rhee et al., 2012; Skafida et al., 2022; Tajima & Harachi, 2010), another six studies reported on adults (Gilligan & Akhtar, 2005; Kimber et al., 2015; Maker et al., 2005; Rai et al., 2022; Sunley et al., 2020; Widanaralalage et al., 2025), three studies reported on adolescent/youth (Black et al., 2013; Lee et al., 2014; Skårdalsmo Bjørgo & Jensen, 2015), and the remaining study reported parent and support workers (Fisher, 2013) (Table 1: Characteristics of studies included in the review). Among included studies, half of them reported physical violence, whereas six reported physical, sexual, and emotional violence and neglect. Regarding the prevalence of child abuse reported, physical violence ranged from 23 to 78%, while emotional abuse, sexual abuse, and neglect ranged from 5.2 to 19.1%, 3.9–13.7%, and 4.7–29.6%, respectively (Table 1: Characteristics of studies included in the review).

**Table 1:** Characteristics of studies included in the review

Authors & Year	Country of Origin	Types of Abuse Studied	Target Population	Study Type	Aim / Purpose	Population / Sample Size	Study Tool Used	Cut Point Used	Key Findings
Lee et al.,^2014^)	USA/ South Korea	Physical abuse, psychological abuse, neglect, sexual abuse	Adolescent/youth	Quantitative study	Comparison of within and between ethnic group differences on what constituted child maltreatment and its severity	62 European American, 26 Korean America and 62 Korean university students were included.	The item from the Cross-Cultural Definitions of Abuse in Families survey:	Participants described the type of abuse they consider serious and mentioned their own perception regarding its severity as per their culture.	Fewer American with European background than Koreans mentioned psychological aggression as an extremely abusive type. European Americans placed greater emphasis on physical aggression, whereas Koreans focused more on psychological aggression and neglect.
Skafida et al., (2022)	Scotland	**Physical abuse (smacking**)	Children and or their parents	Quantitative	1. To assess the extent to which children who experience IPV also have direct experiences of parental aggression. 2. To explore if children of mothers who have experienced IPV are more likely to be smacked or slapped by their parent	5217 participants and or their family were included.	ever slapped or smacked by their parents.	Yes/No.	Boys are likely to be smacked in homes where language other than English is spoken while girls are 19% likely to be smacked by their mother.
Sunley et al., (2020)	Netherland	Physical, sexual abuse, psychological abuse, emotional neglect.	Adults aged 18–70 years	Quantitative	To examines ethnic and sex differences in rates of child maltreatment and depressed mood in adulthood. To investigates whether the association between child maltreatment and depressed mood in adulthood is influenced by ethnicity and sex.	22,551 people aged 18–70 years.	Maltreatment was measured using a self-reported version of the 4-item NEMESIS childhood trauma scale.	Experiencing any one of the abuses before 16 years of age was considered to have maltreatment.	African Surinamese were reported to have higher prevalence of overall maltreatment (35.2%), high rate of psychological abuse (19.1%), physical abuse (22.3%), Sexual abuse (13.7%) and neglect (24.5%). Significantly more women reported sexual violence than man. Ghanaians who reported physical abuse in childhood were the only ethnic group with significantly increased odds for depressed mood, with the same being true for Moroccans who experienced sexual abuse in childhood are more likely to have depressed mood.
Fisher, (2013)	Australia	**Physical abuse (smacking)**	Parents and support workers (adult)	Qualitative.	To explore the interrelationships between changed and changing cultural norms and expectations around gender and family roles and responsibilities, and DV following settlement in Perth, Australia.	A total of 78 individuals participated in the research.	NA	NA	Inability to discipline children, as they used to do in Africa, lead to a lack of respect from their kids, and loss of parental authority in Australia. Participants express their dissatisfactions with influences from the government in dealing with their family and complained inability to control their family including lack of power to discipline their children.
Black et al., (2013)	USA	psychological aggression, physical assault, neglect, and sexual abuse	Iraqi refugee adolescents	Quantitative	To explore the relationships between exposure to violence in the community, school, home and dating relationships among Iraqi American youth	196 Iraqi youth (106 males and 90 females) in grade 5–12.	Parent–Child Conflict Tactics Scale derived from the Conflict Tactics Scale.	reporting any of any of the abuses type is considered as experiencing abuse	Nine (4.7%) of the youth reported being a victim of sexual abuse. 26% report being the victim of acts of neglect. Almost 18% (17.9%; *n* = 35) of them had experienced severe child abuse. No gender differences were found in reported acts of regular or severe child abuse. Physical child abuse experience is significantly associated with having peers with dating violence
Kimber et al., (2015)	USA	Emotional abuse, physical abuse, sexual abuse, physical neglect.	Adult aged 20 years and above	Quantitative	To examine the associations between immigrant generational status, child maltreatment, intimate partner violence and acculturation (i.e., the extent to which an individual adopts the values, language and attitudes of a new culture)	34,653 USA residents aged 20 years and above.	Conflict tactic scale and child trauma scale were used to assess child abuse	Experience any one of abuse components was considered as experiencing the abuse	Physical abuse ♣ 17.8% (3rd generation) ♣ 17% (2nd generation) ♣ 14.8% (1st generation) Emotional abuse ♣ 8.4% (3rd generation) ♣ 7.1% (2nd generation) ♣ 5.2% (1st generation) Sexual abuse ♣ 10.9% (3rd generation) ♣ 9.8% (2nd generation) ♣ 7.3% (1st generation) Neglect ♣ 23.3% (3rd generation) ♣ 22.9% (2nd generation) ♣ 29.6% (1st generation) Overall child abuse ♣ 37.3% (3rd generation) ♣ 35.7% (2nd generation) ♣ 38.2% (1st generation) After adjusting for the confounders, immigrant generational status was not associated with IPV perpetration or victimisation among respondents with a history of childhood maltreatment.
Skårdalsmo et al., (2015)	Norway	Physical violence	Adolescent aged 13–19 years	Qualitative study.	To explore early life experiences of unaccompanied refugee minors who have disclosed experiences of interpersonal violence and physical abuse	34 unaccompanied children seeking asylum in Norway before the age of 15 years	Were asked to describe abuse they had experienced before 15 years of age.	NA	Most of the perpetrator’s violence against children were father/ a person with father role. Participants described dominating role of man in deciding family matter including child discipline and stressed the diminished role of women in decision making in their culture. Participants mentioned that abuse is systematic and common in their countries but approximately half of the youth displayed some positive feeling about the perpetrators blaming themselves for the abuse.
Gilligan and Akhtar (2005)	UK	Women were invited to discuss on insert booklet reporting on “Sexual abuse” that was read out by facilitator. The insert was about child sexual abuse, the decision of the mother not to disclose to protect honour of her family	Adult Asian women ( Pakistani, Indian, Bangladeshi)	Qualitative	To contribute to understanding of issues relating to Asian culture and child sexual abuse.	130 Asian women aged 20 to 60 years from Urdu/ Punjabi-, Bangla-, Pushto- and Gujarati-speaking communities in the UK	NA	NA	The finding has indicated the influence culture has on how people behave, detailing the concept of izzat (honour/respect), haya (modesty) and sharam (shame/embarrassment). Participant mentioned that “the community will keep sexual abuse undercover because of izzat. Such cases are not discussed so easily and openly”. Other participant” It’s difficult to go to someone outside including services for help as this would show that family in a bad light and it could also get out in the community bringing shame to the family”. Another participant “It won’t be easy to talk to people about this kind of things, only with certain members of the family, i.e., those from the same generation and in certain contexts such as when it takes place. ……it’d be difficult to talk to the elder generation about this”.
Kohli and Fineran (2020)	USA	child abuse and neglect	Immigrant latino father	Qualitative	To study the lived experiences of child abuse and neglect for immigrant Latino fathers whose children are involved in the child protection process	9 immigrant Latino father whose children were in the child protection process. 3 were from El Salvador, 2 from Guatemala, and 1 each from Cuba, Costa Rica, Chili and Puerto Rico.	NA	NA	Participants shared that the use of strict disciplining strategies to punish children (e.g., hitting/punching, demanding them to do work beyond their ability, not providing basic needs) is the Latino way of raising children. The Puerto Rican participant summarized disciplining children, “But that’s life, that’s how you raise kids. That’s the Hispanic way…there would be red marks, maybe a bruise or two, but never anything serious.” The participant from Guatemala shared “the culture over there is like, if you touch something that you’re not supposed to, you get a spanking and they would lock me in a room.” Participants mentioned their experience of trauma and fear as a result of harsh discipline they experienced during their childhood, and mentioned how they are struggling with emotion such as anger that resulted from abuse during their childhood.
Degni et al., (2006)	Finland	**Corporal punishment**	Somali parents (adult)	Qualitative study	To explore the Somali parents’ experiences in bringing up children in Finland and identify the social-cultural changes that have and are continuing to take place within the migrant-household	21 respondents chosen from two cities (Helsinki and Turku).	NA	NA	Both men and women participants believe that corporal punishment is acceptable and valid parenting culture among Somali community although women indicated some reservation regarding corporal punishment, mentioning the need to look for alternative approach without corporal punishment. They also mentioned that in Somali culture, father is the head of the family and had the authority, and should be naturally respected, while children has natural duty to respect their parents. Participants also indicated Somalis dissatisfaction with parenting practices in Finland reporting conflicting parenting style with their owns.
Tajima & Harachi, (2010)	US	**Physical discipline**	Southeast Asians parents(adult).	Quantitative study	Investigate the parenting beliefs and physical discipline practices of Southeast Asian parents in the context of immigration and acculturation, by examining factors related to breaking the intergenerational cycle of physical discipline	308 parents (153 Cambodian, 155 Vietnamese).	Item from conflict tactic scale was used to assess spanking/physical abuse	Reporting any act of physical abuse was considered as experiencing abuse.	As high as 74% of participants reported ever having used physical punishment with their children. The finding indicated that spanking is significantly common in among Vietnamese compared to Cambodians whereas hitting with object is common among Cambodians than Vietnamese. Cambodians were more likely to emphasize obedience compared to Vietnamese respondents in raising their children. Vietnamese were significantly more likely to emphasize helping others than were the Cambodian parents in raising their children. Acculturation is important factors in reducing rate of physical discipline among Cambodian but not for Vietnamese. Regrading gender, breaking cycle of physical discipline is less common for male compared to women. Parent’s own childhood history of physical discipline more than doubled the likelihood of using physical discipline with their own child.
Maker et al., (2005)	USA	Physical abuse	Women of Latina, east Asia, south east Asian and middle east origin were participated	Quantitative	To examine the prevalence, characteristics, beliefs, and demographic predictors of parent-child physical violence among South Asian, Middle Eastern, East Asian, and Latina women in the United States	251 women (mean age of 25 years)	Eight items from Finkelho’s questionnaire were used to assess childhood physical violence that occur before the age of 16 years.	Reporting any of the physical violence is considered as having physical violence.	73% of the South Asian and Middle Eastern group, 65% of the East Asian group, and 78% of the Latina group reported experiencing at least one type of physical abuse before the age of 16. For South Asian and Middle Eastern, years father and mother had lived in USA was positively correlated with physical abuse while the correlation is negative for East Asian. For Latinas, only the number of years the father had been in the United States was significantly and negatively correlated with physical abuse. Among east Asian and Latinas, experiences of physical abuse were positively and significantly correlated to higher levels of belief that mothers and fathers have the right and privilege to hit their children
Dettlaff & Johnson, ( 2011)	USA	Physical abuse, sexual abuse, emotional abuse, neglect	Hispanic/Latino Children who were subjects of reports of maltreatment to child welfare agencies	Quantitative	The goals of this study are to establish the national prevalence of immigrant and U.S. born Latino children who come to the attention of child welfare systems in the United States; -to expand our knowledge regarding the role of nativity in child maltreatment patterns among Latinos; and to determine if disparities in child maltreatment patterns and risks exist among Latino families	947 Latino children were included.	Types of abuse directly extracted from the case files	NA	Immigrant Latino children were nearly three times as likely to be victims of physical abuse than U.S. born Latino children (75.7% vs. 27.8%). U.S. born Latino children were significantly more likely than immigrant Latino children to be victims of emotional abuse (24.2% vs. 0.2%). The finding indicated no significant difference in overall abuse among Latino children born in USA and outside USA. Furthermore, male constitute large proportion of foreign-born immigrants (58.6%) reported abuse compared female (41.4%).
Park, (2001)	USA	Child physical abuse	First generation immigrant women who have children below 18 years were interviewed.	Quantitative	Explores Korean immigrant mothers’ attitudes toward child physical abuse based on an ecological perspective.	144 Korean immigrant mothers with children below 18 years old participated in this study	Item from Conflict Tactics Scale were used to assess abuse.	Reporting any physical abuse	Mothers who report that children are valued highly in Korean culture are less likely to be negative about abusive behaviours toward children. Mothers who have a son and have high family acculturation conflict, and report experiences of stress because of their immigrant status and discrimination are more likely to use physical aggression toward children when conflict occurs between them and their children. Mothers who have high family acculturation conflicts, feel positively about corporal punishment as a part of Korean society, and report experiencing discrimination because they are immigrants are more likely to have positive beliefs about use of physical punishment.
Rhee et al. (2012)	USA	Physical, sexual, emotional abuse, neglect with victims age ranging from 1 month to 17 years.	124 Vietnamese children case files from child protective services	Quantitative	To fill the gap in the knowledge base of child maltreatment among immigrant Vietnamese families,	124 Vietnamese children case files from child protective services	Types of abuse directly extracted from the case files	NA	The perpetrator’s length of residence in the US is also significantly associated with severity of abuse, indicating that relatively new immigrants are more likely to abuse their children more severely. However, the relationship between gender of the perpetrators and severity was not significant.
Renzaho et al., (2011)	Australia	Physical abuse	A total of 10 focus group discussions (five with parents and five with 13–17-yearold children; *N* = 85 participants)	Qualitative	To report on a study that set out to document parenting styles among African migrants and to assess how intergenerational issues related to parenting in a new culture impact on family functioning and the modification of lifestyles	85 participants of Ethiopian, Sudani and Somali origin in Australia	Physical discipline was asked directly	NA	Parents described the existence strong control regarding their children’s behaviour and their access to social environment. Family also mentioned the disciplining their if they are in short of their family’s expectations. Furthermore, the relationship between the family and children are often gender specific with father considered as head of the family who make final decision regarding family matter. Father often disciplines the kids while mother nurture them.
Rai et al., (2022)	USA	Domestic violence perceptions, including attitudes toward interpersonal violence and physical punishment of children (spanking, hitting children with a belt).	South Asian immigrant men and women residing in the United States.	Quantitative (cross-sectional study)	To examine domestic violence perceptions and correlates among South Asian immigrants, including attitudes toward violence within families.	South Asian (*N* = 468) of 18 years or older, and residing in the US	Modified Perceptions of and Attitudes Toward Domestic Violence Questionnaire-Revised (PADV-R). Child punishment was measured within the Attitudes Toward the Use of Interpersonal Violence Scale, including the subscale Physical Punishment with Children.	PADV-R used a 6-point Likert scale. Mean scores **≤** 3.51 = agree, ≥ 3.52 = disagree	♣ Participants generally did not support physical punishment of children. Mean scores for the Attitudes Toward the Use of Interpersonal Violence Scale were above 3.52, indicating disagreement with using force for family matters and children. ♣ Gender-role attitudes were significantly associated with attitudes toward interpersonal violence, including child punishment. Participants with more progressive/liberal gender-role attitudes were less likely to condone violence to solve family matters, whereas more conservative gender-role attitudes were associated with greater acceptance of interpersonal violence.
Widanaralage et al., (2025)	UK	Childhood sexual abuse, childhood sexual violence, rape, sexual assault, assault by penetration, forced-to-penetrate experiences among males	South Asian ( Pakistani, Indian, Bangladesh, Sri Lankan) male survivors of childhood sexual abuse/sexual violence living in the UK	Qualitative study using Interpretative Phenomenological Analysis (IPA) with semi-structured	To explore how South Asian cultural norms shape disclosure and help-seeking among adult male survivors of childhood sexual abuse in the UK, and to understand barriers to disclosure, support-seeking, and engagement with criminal justice services.	11 South Asian male survivors of childhood sexual violence currently living in the UK.	No standardized violence measurement instrument was used. Data were collected through semi-structured qualitative interviews exploring experiences of sexual violence, disclosure, cultural factors, and support-seeking.	NA	Traditional South Asian values of izzat (honour) and sharam (shame**)** strongly influenced survivors’ experiences. Cultural expectations that men should be strong, resilient, and successful discouraged disclosure and help-seeking. Survivors feared appearing weak, vulnerable, or unmanly. Cultural taboos surrounding sex and mental health further inhibited recognition of abuse, disclosure, and access to support. Family reputation was often prioritized over survivor wellbeing.

### Cultural Beliefs, Disciplinary Practices, and Acculturation as Contexts for Child Abuse

The current review has indicated variation in the level of abuse across immigrant communities, as each and every immigrant community has its own distinct culture and understanding of violence. For example, in a study from the Netherlands, African Surinamese were reported to have a higher prevalence of overall maltreatment (35.2%), a high rate of psychological abuse (19.1%), physical abuse (22.3%), sexual abuse (13.7%) and neglect (24.5%) compared to people from Turkey, Morocco, Ghana and even from the Netherlanders (Sunley et al., 2020). Similarly, a wide variation of physical abuse during childhood was reported among people from South Asian and Middle Eastern (73%), East Asian (65%), and Latina (78%) cultural backgrounds (Maker et al., 2005). The findings also indicated the existence of diverse understanding regarding the severity of abuse. For example, participants from Korean backgrounds placed greater emphasis on psychological aggression or neglect while people from European background give more emphasis on physical aggression (Lee et al., 2014).

The acceptability and frequent use of corporal punishment were reported across cultural groups. From the perspectives of participants from Somalia (Filio Degni et al., 2006), Liberia, Sierra Leone, Ethiopia, Sudan (Fisher, 2013), Afghanistan, Eritrea, Sri Lanka (Skårdalsmo Bjørgo & Jensen, 2015), El Salvador, Guatemala, Chile, Costa Rica, and Cuba (Kohli & Feneran, 2020) ; physical punishment was a common method of discipline in their cultures. Park et al. (2001) also found that Korean mothers who reported that children were highly valued in their culture were less likely to perceive abusive behaviours negatively, further reflecting cultural acceptance of strict disciplining methods. However, the included studies showed differences in the nature of violence across communities. For example, in a study from the USA, spanking is more common in immigrants with Vietnamese backgrounds compared to Cambodians, while hitting children with objects is common in Cambodians compared to Vietnamese (Tajima & Harachi, 2010). Similarly, Marker et al. (2005) found that hair pulling and slapping were more often experienced by Latina, South Asian, and Middle Eastern participants than East Asian participants, while spanking with a belt, switch, or whip was more often experienced by East Asian and Latina participants than South Asian and Middle Eastern participants (Maker et al., 2005; Rai et al., 2022). Another study reported that hitting, punching, demanding children to work beyond their ability, locking them in a room, and not providing basic needs as a way of punishing children when they misbehave in Latinas culture (Kohli & Feneran, 2020), while withholding food and smacking were also practiced among East African immigrants in Australia (Renzaho et al., 2011).

The review also indicated that disclosure of abuse is a major challenge among immigrant communities. For example, studies involving participants of Pakistani, Indian, Bangladeshi, and Sri Lankan backgrounds in the UK found that honour- and shame-based cultural norms, traditional gender expectations, and taboos surrounding sex hindered the disclosure of child sexual abuse and help-seeking among both female and male survivors. Family reputation was often prioritised over survivor wellbeing in these communities (Gilligan & Akhtar, 2005; Widanaralalage et al., 2025). In addition, blaming oneself for the violence among the victims and acceptance of violence is reported in a qualitative study from a sample of unaccompanied refuge minors from Norway (Skårdalsmo Bjørgo & Jensen, 2015).

Acculturation and length of stay in the host country are also found to be related to child abuse in most studies (Kimber et al., 2015; Lee et al., 2014; Maker et al., 2005; Rhee et al., 2012; Tajima & Harachi, 2010). Some studies reported a decrease in the risk of child abuse as perpetrators spent more years in the host country (Dettlaff & Johnson, 2011; Rhee et al., 2012). In contrast, other studies indicated that perpetrator’s gender and country of origin may shape the relationship between length of stay in the host country and child abuse (Maker et al., 2005; Tajima & Harachi, 2010). For example, Maker et al. (2005) found that the length of time that mother and father had lived in the U.S. was positively associated with physical abuse in Asian and Middle Eastern sample, but negatively associated with physical abuse in a sample of Eastern Asian participants (Maker et al., 2005). Similarly, Tajima and Harachi (2010) found that stronger acculturation to the U.S. decreased Cambodian respondents’ likelihood of using physical discipline, but not for Vietnamese respondents (Tajima & Harachi, 2010). Another study that reported on child nativity revealed that immigrant Latino children were nearly three times as likely to be victims of physical abuse than U.S.-born Latino children but were significantly less likely than U.S.-born Latino children to be emotionally abused (Dettlaff & Johnson, 2011). In addition, inter-generational differences in types of abuse experienced were reported by Kimber et al. (2015), with first-generation immigrants being less likely than third-generation immigrants to report a history of sexual and emotional abuse but more likely to report a history of physical neglect (Kimber et al., 2015).

### Gender Roles, Family Power Dynamics, and Disclosure of Abuse

In many of the communities within the scope of the current review, gender and family roles are guided by patriarchal beliefs and values. Fathers had more authority than mothers in the household as the head of the family and the breadwinner (Filio Degni et al., 2006; Fisher, 2013; Renzaho et al., 2011; Skårdalsmo Bjørgo & Jensen, 2015). Conversely, mothers’ expected role was to take care of and raise their children (Filio Degni et al., 2006) and/or provide emotional support for the family (Fisher, 2013; Renzaho et al., 2011). Interestingly, mothers were also found to be less likely to authorize physical punishment (Filio Degni et al., 2006), and children were also less likely to have negative feelings towards their mothers for perpetrating physical punishment (Skårdalsmo Bjørgo & Jensen, 2015). In some communities, such as South Asian communities, attitudes towards child punishment varied according to individual gender-role beliefs, with more progressive gender-role attitudes associated with lower acceptance of interpersonal violence, including child punishment, and more conservative attitudes associated with greater acceptance of violence (Rai et al., 2022).

Regarding victimization, the review indicated no significant gender difference in victimization regarding overall abuse among males and females (Black et al., 2013; Sunley et al., 2020), although one study reported a higher rate of abuse among boys than girls (Skafida et al., 2022). However, specific to each aspect of child abuse, females are more likely to experience sexual abuse (Kimber et al., 2015; Sunley et al., 2020), whereas males are likely to experience physical abuse (Kimber et al., 2015).

### Impacts of Childhood Abuse

Three of the included studies described the impacts of the childhood experience of domestic violence on victims’ well-being. For example, a study from the Netherlands indicated the role of cultural backgrounds in relation to abuse impacts, reporting that Ghanaians who reported physical abuse during childhood were the only ethnic group with significantly increased odds for depressed mood, while Moroccans who experienced sexual violence during childhood are more likely to experience depression (Sunley et al., 2020). In a study involving Immigrant Latino fathers, harsh disciplining during their childhood caused trauma and fear, and that they still struggle with negative emotions resulting from their childhood experience of abuse (Kohli & Feneran, 2020). Similarly, a study that employed Somali mothers and fathers from Finland reported the effect of corporal punishment in reducing children’s self-esteem and causing aggression (Filio Degni et al., 2006).

The relationship between personal experience of childhood abuse and the perpetration of violence during adulthood was reported in three studies. In a study that employed Iraqi youths from the USA, it was reported that experiencing physical child abuse is associated with knowing friends who experienced dating violence (Black et al., 2013). However, another study from the USA found no significant association between a history of childhood maltreatment and intimate partner violence perpetration or victimization (Kimber et al., 2015). A USA study found that Cambodian and Vietnamese parents who were disciplined during childhood were more likely to use physical discipline with their child, indicating intergenerational transmission of physical discipline (Tajima & Harachi, 2010).

## Discussion

This scoping review attempted to synthesize evidence that reported on cultural issues in relation to childhood experiences of family violence and provide evidence regarding how these cultural issues impact the outcomes of abuse. The findings from this review indicated various culturally driven beliefs and attitudes that need to be addressed in tackling violence against children.

In this review, although the rate of child abuse is different among participants from different cultural backgrounds, overall, substantial levels of child abuse were reported across immigrant communities. This might be attributed to different cultural beliefs that are likely to contribute to child abuse. For example, having a belief about the right and privilege to hit children among parents was associated with an increased likelihood of child physical abuse in a sample of South Asian, Middle Eastern, and Latinas participants (Maker et al., 2005) while a study of participants from Korean background has indicated a positive attitude towards child physical punishment among women who have a belief that children are valued in their culture (Park, 2001).

This review identified that gender and family roles are often guided by patriarchal beliefs and values among immigrant communities. The patriarchal gender role is common among immigrants predominantly from African backgrounds, with a strong emphasis on the male as the head of the house and the male having greater power and authority over family matters, including child discipline (Filio Degni et al., 2006; Fisher, 2013; Renzaho et al., 2011). For example, fathers have the authority to control their children and make decisions regarding family matters, including child punishment, while children are expected to obey and be respectful to their families (Filio Degni et al., 2006; Renzaho et al., 2011). The existence of patriarchal gender roles is very problematic for children. A systematic review of studies from Southeast Asia has indicated that in a cultural setting that creates an imbalance of power due to patriarchal gender norms, children are often put in a difficult situation that exposes them to abuse (Fry et al., 2020). Even in a country where child abuse is considered illegal, children who grow up in a patriarchal family might live in persistent abuse as they may be conditioned to tolerate the abuse, potentially preventing them from seeking support, as indicated in previous review studies (Abi Rached et al., 2021; Fry et al., 2020; Pereira et al., 2020). This underscores the need for interventions that move beyond simply identifying abusive behaviours to addressing the underlying cultural beliefs and norms that may sustain violence against children.

In this review, we further investigated whether the existence of patriarchal gender norms within the immigrant community is practically related to the difference in abuse among boys and girls. However, the finding from the review did not indicate gender differences in victimization regarding overall abuse (Black et al., 2013; Sunley et al., 2020), although one study reported a higher rate of abuse among boys than girls (Skafida et al., 2022). Interestingly, when a specific aspect of child abuse was investigated, girls were more likely to experience sexual abuse (Kimber et al., 2015; Sunley et al., 2020), whereas boys were likely to experience physical abuse (Kimber et al., 2015). This finding is consistent with previous review indicating that girls are more frequently identified as victims of child sexual abuse than boys (Behl et al., 2003). The high prevalence of sexual abuse in this review might be related to the existence of attitudes and gender roles that view girls/females as more vulnerable or submissive. Additionally, boys may be seen as more physically powerful and more able to defend themselves against sexual violence. In addition, the higher rate of physical violence among boys may be related to the fact that boys are more likely to be involved in physical altercations and conflicts, as reported in previous studies (Nilsson, 2017; Thompson et al., 2004). These dynamics highlight how gender norms intersect with cultural beliefs to influence both vulnerabilities to abuse and responses to victimization. Addressing childhood abuse in immigrant communities therefore requires explicit attention to gender relations and power imbalances, rather than treating culture as a homogeneous or static construct.

Evidence has indicated a strong link between cultural values and beliefs and child abuse, reporting the wide variety of child abuse practices and acceptability from culture to culture (Stoltenborgh et al., 2011). In line with this, the current review found that disciplinary practices within immigrant communities are shaped by culturally embedded norms regarding parental authority, child obedience, and acceptable forms of correction (Maker et al., 2005; Tajima & Harachi, 2010). These culturally patterned practices reflect broader socialization processes that are distinct to each community, through which disciplinary norms are learned, reinforced, and transmitted across generations, rather than isolated parental behaviors, highlighting how abuse may be normalized within certain contexts despite legal and social prohibitions in host countries. Such variation underscores the importance of understanding child abuse within its cultural and structural context, rather than applying uniform interpretations across diverse communities.

Acculturation, the process of adapting to a new culture, affects child abuse differently depending on a specific cultural context. The current review showed a decrease in the risk of child abuse with the number of years perpetrators had lived in the host country due to possible acculturation (Dettlaff & Johnson, 2011; Rhee et al., 2012). Acculturation might bring about more positive changes by improving access to resources and supporting systems that help prevent abuses. Furthermore, adapting to a new culture might lead to changes in cultural beliefs, including shifting away from corporal punishment as a means of child discipline. However, it is worth noting that the relationship between years spent in the host countries and child abuse is not always linear. For example, a USA study has indicated stronger acculturation to the U.S. decreased Cambodian respondents’ likelihood of using physical discipline, but not for Vietnamese. Therefore, the conflicting relationship between acculturation and child abuse might be related to differences in cultural context, individual experience, and the availability of resources and support systems for the families of immigrants in the host countries.

Evidence has indicated that children who experienced childhood abuse often experience different behavioural and mental health problems such as abusive behaviour, chronic stress, anti-social behaviours, depression, and drug abuse (Al Odhayani et al., 2013). In agreement with this, the current review has indicated the major consequences of child abuse among immigrant communities, including depression, anger, and low self-esteem. This indicates the need to provide culturally sensitive services, including psycho-social services that address abused children’s specific psycho-social needs. Furthermore, this review reported that victims of child abuse are more likely to perpetrate physical abuse against their children and more likely to know someone who perpetrated dating violence indicating possible intergenerational transmission of child abuse. However, further research is needed to investigate factors that contribute to the intergenerational transmissibility of violence across immigrant communities to better understand and design interventions that break intergenerational cycles of abuse against children.

A number of cultural issues were speculated to obstruct the disclosure and help-seeking behaviors in the context of child abuse, most specifically childhood sexual abuse (Shafe & Hutchinson, 2014). Many aspects of childhood sexual abuse, including the act itself, and the perceptions of family, neighbors, and friends, contribute to feelings of shame, although the level differs from culture to culture. In cultural contexts where family honour and reputation are prioritised over the wellbeing of individual victims, survivors may experience difficulties disclosing sexual abuse (Fontes & Plummer, 2010). This is reflected in the current review which identified cultural taboos related to family honour, modesty, and shame as barriers to disclosure and help-seeking among South Asian women and men who experienced child sexual abuse in the UK (Gilligan & Akhtar, 2005; Widanaralalage et al., 2025).

### Strengths and Limitations of the Study

Due to the nature of the study and the heterogeneity of the included studies, pooling the findings of this review was not possible. Furthermore, most of the included studies were from the USA, which may affect the generalizability of the findings. Regardless of these limitations, the current review provided evidence on the role of culture in child abuse and underlined the potential consequences of abuse among immigrant communities. In addition, using thorough search terms without limiting to the date of publications and including qualitative and quantitative studies in the review improves the discoverability of all potential articles.

### Implications of the Study

The review highlighted substantial levels of child abuse across immigrant communities, although the nature and acceptability of violence varied across cultural groups. As the number and geographical distribution of the studies included in this review were insufficient to capture all possible roles of cultures, further studies are needed to understand the role of culture in child abuse. In addition, the current review reported different culturally driven attitudes that contribute to child abuse and inhibit disclosure and support-seeking among abuse victims.

Providing culturally sensitive services is very important to encourage disclosure and break the cycle of violence in the community (Ettinger, 2022). In the current study, people with immigrant backgrounds raised their concerns and dissatisfaction regarding the existing child welfare system that took away their kids for their safety. Apart from educating people about child abuse, programs addressing child abuse in immigrant communities should embed cultural sensitivity at the core of assessment, intervention, and care strategies, while critically reflecting on how cultural norms are engaged in practice. Culturally sensitive approaches should aim to understand the values underpinning family practices through partnership with communities, as prevention efforts that do not involve communities may be perceived as punitive, culturally insensitive, or threatening, potentially entrenching mistrust and reducing engagement with support services. Similar community-partnership approaches have been shown to be effective in other culturally distinct populations, such as Aboriginal communities, highlighting the broader value of culturally grounded approach and offers transferable lessons for services working with immigrant communities (Alaggia, 2010). Furthermore, improving parenting skills through training and education and creating employment opportunities may help families integrate into the dominant culture and reduce economic strain and child abuse.

## Conclusion

The review identified substantial levels of child abuse among immigrant communities. Different cultural beliefs and gender-related norms were reported to influence the acceptability of child abuse and help-seeking behaviours among victims. To combat this issue, it is crucial to address cultural beliefs that condone child abuse through education and training. Additionally, the provision and enhancement of culturally sensitive services are vital to promote disclosure, prevent negative mental well-being, and break the cycle of violence within these communities.

## Supplementary Information

Supplementary Material 1.

## Data Availability

The data related to the findings of this study are all included in this review.
