# Supplementary material for: The Influence of Cultural And Gender Roles On Child Abuse Among Immigrant Communities: A Scoping Review
Source: J Child Adolesc Trauma. 2026 Jul 1;19(4):1943–62. doi: 10.1007/s40653-026-00936-w (PMC13737213; doi:10.1007/s40653-026-00936-w)
Supplement: Supplementary file 1 [file 40653_2026_936_MOESM1_ESM.docx]

**Cover letter**

**Dear Editor**,

Thank you for the opportunity to revise our manuscript, "The Influence of Cultural and Gender Roles on Child Abuse Among Immigrant Communities: A Scoping Review."

We have carefully considered all reviewer comments and revised the manuscript accordingly. In addition, we updated the literature search and identified two additional eligible studies, which have been incorporated into the review. All corresponding revisions have been reflected throughout the manuscript, tables, and references.

A detailed point-by-point response to the reviewers' comments is provided in the accompanying response document. We have uploaded both a tracked-changes version and a clean version of the revised manuscript.

We appreciate the time and effort of the editor and reviewers and hope that the revised manuscript is now suitable for publication in the Journal of Child & Adolescent Trauma.

Yours sincerely,
